# Changes in antidiabetic prescription patterns and indicators of diabetic control among 200,000 patients over 13 years at a single institution in Japan

**DOI:** 10.1186/s13098-016-0187-8

**Published:** 2016-11-10

**Authors:** Kazutoshi Fujibayashi, Michio Hayashi, Hirohide Yokokawa, Toshio Naito

**Affiliations:** 1 Department of General Medicine, School of Medicine, Juntendo University, 3-1-1, Hongo, Bunkyo-ku, Tokyo 113-8421 Japan; 2 Endocrinology and Metabolism Division, NTT Medical Center Tokyo, 5-9-22 Higashi-Gotanda, Shinagawa-ku, Tokyo 141-8625 Japan

**Keywords:** History, Drug therapy, Control, Numerical data

## Abstract

**Background:**

We examined the long-term changes in the management of diabetes at a single institution in Japan.

**Methods:**

Two repeated cross-sectional studies and a retrospective cohort study were conducted among patients who visited our institution between 2001 and 2013. We examined the changes in glycated hemoglobin (HbA1c) and glycated albumin levels, the prescription frequencies, and the daily doses of each antidiabetic agent among patients treated regularly for diabetes during the 13-year study period. The trends in control and treatment parameters were analyzed using Spearman’s rank correlation coefficient.

**Results:**

In the first repeated cross-sectional studies, 200,298 patients had their glucose metabolism indicators measured, and diabetologists prescribed medications to 193, 445 patients. Of these, 170 patients were included in the retrospective cohort study. The patients’ diabetic control tended to improve over the study period. The mean HbA1c level improved from 7.9 to 7.6% (from 63 to 60 mmol/mol) (rs = −0.11, p < 0.01) in the cross-sectional study, corresponding to a change from 8.2 to 7.7% (from 66 to 61 mmol/mol) (rs = −0.22, p < 0.01) in the retrospective study. The mean GA level improved from 22.7 to 20.7% (rs = −0.13, p < 0.01) in the cross-sectional study and from 23.5 to 21.5% (rs = −0.14, p < 0.01) in the retrospective study. Over the study period, prescription frequencies and daily doses of antidiabetic agents changed as treatment guidelines were altered.

**Conclusions:**

The present study revealed a tendency toward long-term improvements in diabetic control, with changes in the prescription patterns consistent with research and guideline evidence.

**Electronic supplementary material:**

The online version of this article (doi:10.1186/s13098-016-0187-8) contains supplementary material, which is available to authorized users.

## Background

The incidence of diabetes is rising globally. As of 2013, 382 million people had the disease, with that number expected to increase to 592 million by 2035 [1]. In Japan, 16.2% of men and 9.2% of women either have or are strongly suspected of having diabetes [2]. As the number of patients with diabetes increases, the associated medical costs rise in tandem. In 2013, global health expenditure due to diabetes was estimated to be USD 548 billion. Moreover, those costs are expected to increase to USD 627 billion by 2035. Consequently, diabetes is considered a serious health problem worldwide [3].

Glycated hemoglobin (HbA1c) levels are affected by anemia, age, and other factors, and when used in isolation, they are not very accurate for monitoring diabetes in patients with conditions such as impaired renal function or in those who are pregnant [4–6]. Glycated albumin levels, which reflect blood glucose levels over the previous two weeks and are useful in managing diabetes with chronic renal dysfunction [7–9]. Therefore, glycated albumin levels are used as an indicator of diabetes management in Japan.

In 2002, intensive therapy and lowering of HbA1c were effective in preventing diabetic complications [10]. A diagnosis of metabolic syndrome that was caused by insulin resistance increases was focused on in 2005 [11]. In 2006, biguanide was designated as the first-line drug for diabetes treatment, and thiazolidine was added as the second-line drug [12]. In 2007, the importance of postprandial hyperglycemia management was shown [13]. In 2012, incretin-related drugs were added as second-line drugs [14]. In addition, a patient-centered approach was recommended.

Although the diabetes management guidelines have been updated frequently, changes in the overall treatment of diabetes and changes in the diabetes management of each patient remains unclear. Repeated cross-sectional analyses can observe overall tendencies of all patients. However, such study designs have some limitations regarding the observation of the course of each patient. In addition, retrospective cohort analyses have the opposite characteristic. Therefore, we conducted two large-scale repeated cross-sectional studies and a single long-term retrospective study to address these problems.

Our aims were to characterize the effects of evolving diabetes management guidelines by examining long-term changes in prescribing antidiabetic medications and changes in diabetic control parameters.

## Methods

### Study population

This article describes two repeated cross-sectional studies and a retrospective cohort study of patients who visited the Endocrinology and Metabolism Division of the Nippon Telegraph and Telephone Corporation (NTT) Medical Center, Tokyo between January 2001 and May 2013. The Endocrinology and Metabolism Division had between two and five diabetologists at a time during the study period. They treated over 10,000 outpatients and approximately 200 inpatients per year in total in an urban area around Tokyo in Japan. The analyzed information included that of inpatients and outpatients.

First, we performed two repeated cross-sectional studies to examine the changes in diabetes management parameters and prescriptions that occurred between 2001 and 2013. All patient identification (ID) numbers were coded following the data extraction so that individuals could not be identified.

#### The repeated cross-sectional study of diabetes management parameters

**Fig. 1:**
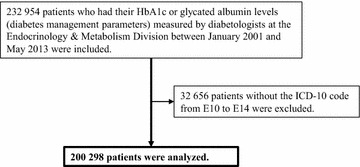
Inclusion and exclusion criteria for the repeated cross-sectional study of diabetes management parameters. *ICD* international statistical classification of diseases and related health problems

First, we extracted the blood test results for patients who had had their HbA1c or glycated albumin levels measured at the Endocrinology and Metabolism Division during the study period, together with the patients’ ID numbers and test dates. The inclusion and exclusion criteria are shown in Fig. 1. The Japan Diabetes Society (JDS) HbA1c values were measured. These values were converted to International Federation of Clinical Chemistry (IFCC) values via National Glycohemoglobin Standardization Program (NGSP) values using the following formulae: HbA1c (NGSP) (%) = {[HbA1c (JDS) (%) × 1.02] + 0.25 (%)} and HbA1c (IFCC) (mmol/mol) = {[10.93 × NGSP (%)] − 23.50 (mmol/mol)} [15, 16]. Next, using International Statistical Classification of Diseases and Related Health Problems (ICD)-10 codes [17], we excluded patients without the following diagnoses: E10, type 1 diabetes mellitus; E11, type 2 diabetes mellitus; E12, malnutrition-related diabetes mellitus; E13, other specified diabetes mellitus; and E14, unspecified diabetes mellitus. To identify changes in diabetic control for the period from 2001 to 2013, we calculated the mean HbA1c levels, glycated albumin levels, or both per year.

#### The repeated cross-sectional study of antidiabetic prescription frequency

**Fig. 2:**
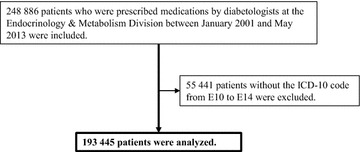
Inclusion and exclusion criteria for the repeated cross-sectional study of antidiabetic prescriptions. *ICD* international statistical classification of diseases and related health problems

The study of antidiabetic prescription patterns was conducted similarly to the study of changes in the indicators of diabetic control. The inclusion and exclusion criteria are shown in Fig. 2. We included patients who were prescribed medication by diabetologists at the Endocrinology and Metabolism Division between January 2001 and May 2013 but excluded patients without ICD-10 codes E10 to E14. To identify trends in antidiabetic medication prescriptions, we calculated the annual prescription rate and annual median daily dose for each antidiabetic medication, based on the prescription records for the study period.

#### The long-term retrospective cohort study of patients with diabetes

**Fig. 3:**
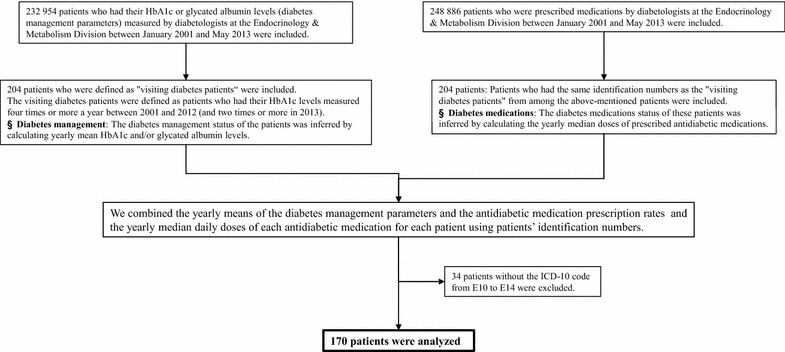
Inclusion and exclusion criteria for the retrospective cohort study of visiting diabetes patients. *ICD* international statistical classification of diseases and related health problems

This study was performed to assess the changes in diabetic control and prescription patterns in a group of patients who continued visiting the hospital for diabetic management from 2001 to 2013. The inclusion and exclusion criteria are shown in Fig. 3. First, we defined patients who had their HbA1c levels measured at least four times a year and at least twice in 2013 as “visiting patients with diabetes.” In general, during diabetes treatment in Japan, HbA1c measurements are obtained once every one to three months, meaning that almost all patients who had their HbA1c levels measured at least four times a year could be considered to have diabetes requiring treatment. From among the visiting patients with diabetes, we then extracted all patients who attended hospital for treatment of their diabetes during the 13 years from 2001 to 2013. We calculated the mean values of each management parameter for each year in each patient who continued to visit the hospital.

We extracted patients, from the repeated cross-sectional study of antidiabetic prescriptions, whose ID numbers matched those of the visiting patients with diabetes. We then determined which antidiabetic medications were prescribed to these patients each year, then calculated the median daily dose of each antidiabetic medication per year per visiting patient. Finally, using the patient ID numbers, we combined the annual means for the diabetic control parameters, the antidiabetic medication prescription rates, and the annual median daily doses of antidiabetic medications. From this combined dataset, we estimated the trends in diabetic control parameters (based on the means per year), the annual prescription frequency per antidiabetic medication (based on the means per year), and the daily doses of antidiabetic medications (based on the medians per year) in patients who continued receiving treatment for diabetes between 2001 and 2013.

### Statistical analysis

In the repeated cross-sectional studies, trends for changes in diabetic control parameters and the changes in daily doses of antidiabetic medications were estimated using Spearman’s rank correlation coefficient. For the control parameters, negative correlation coefficients were interpreted as being indicative of improvements in diabetic control; for the medication usage, positive correlation coefficients were considered to indicate an increase in the daily dose of an antidiabetic medication. Based on the data regarding the patients’ HbA1c and glycated albumin values, participants were divided into four HbA1c categories: <6% (<42 mmol/mol), 6 to <7% (42 to <53 mmol/mol), 7 to <8% (53 to <64 mmol/mol), and ≥8% (≥64 mmol/mol) (derived from the Japanese guidelines) and equivalent glycoalbumin quartiles [18]. The annual changes in each category were evaluated using the Chi square test. Subsequently, trends in the prescription frequencies of antidiabetic medications were analyzed using the Cochran–Armitage test for trend. Annual changes in the mean diabetic control parameters, the median daily antidiabetic medication doses, and the mean antidiabetic medication prescription frequencies were evaluated in the retrospective cohort study using the same methods as for the cross-sectional studies. However, although HbA1c categories were created in the same way, glycated albumin quartiles were based on the mean glycated albumin levels of the retrospective cohort. All calculations were performed with the statistical software JMP Pro version 11 (SAS Institute Inc., Cary, NC, USA). Continuous data are reported as mean ± standard deviation or as median values (inter-quartile range) [range]. p < 0.05 were considered statistically significant.

## Results

### The repeated cross-sectional study of diabetic control

Table 1 shows extracted glycated hemoglobin and extracted glycated albumin data for changes in the parameters of diabetic control observed in the repeated cross-sectional study. An additional file shows this in detail (see Additional file 1). A total of 200,298 patients were included, of whom approximately 70% were men. The HbA1c levels of 199,276 patients were measured between 2001 and 2013, and the glycated albumin levels of 160,191 were measured between 2003 and 2013. The mean HbA1c level tended to decrease from 7.8% (62 mmol/mol) in 2001 to 7.5% (58 mmol/mol) in 2013 (rs = −0.11, p < 0.01). Regarding the four HbA1c categories, the proportion of patients with HbA1c levels >8% (>64 mmol/mol) decreased from 43% in 2001 to 30% in 2013, while the proportion of patients with HbA1c levels of 6 to <7% (42 to <53 mmol/mol) increased from 19% in 2001 to 30% in 2013. Similar to the results for HbA1c, the mean glycated albumin level tended to decrease from 22.7% in 2003 to 20.7% in 2013 (rs = −0.13, p < 0.01). Furthermore, the proportion of patients with glycated albumin levels ≥20.7% decreased from 62% in 2003 to 43% in 2013, while the proportion of patients with glycated albumin levels <18.0% increased from 15% in 2003 to 35% in 2013.

**Table 1:** The extracted glycated hemoglobin and extracted glycated albumin data in the first repeated cross-sectional study

Year	2001	2005	2010	2013	rs*	p value*
Measured HbA1c (n)	10,651	14,342	18,783	7846		
HbA1c (NGSP) (%)	7.9 ± 1.4	7.9 ± 1.4	7.7 ± 1.4	7.6 ± 1.4	−0.11	<0.01
HbA1c (IFCC) (mmol/mol)	63 ± 16	63 ± 16	60 ± 15	59 ± 15		
HbA1c (NGSP) group^a^						
<6% (%)	615 (6)	471 (3)	935 (5)	511 (7)		
≥6% and <7% (%)	1997 (19)	2786 (19)	5194 (28)	2367 (30)		
≥7% and <8% (%)	3457 (33)	5238 (37)	6621 (35)	2585 (33)		
≥8% (%)	4582 (43)	5847 (41)	6033 (32)	2383 (30)		<0.01^#^

Year	2003	2005	2010	2013	rs*	p value*
Measured glycated albumin (n)	8415	12,934	17,681	7463		
Glycated albumin (%)	22.7 ± 5.3	22.5 ± 5.6	21.2 ± 5.5	20.7 ± 5.7	−0.13	<0.01
Glycated albumin quartile^a^						
<18% (%)	1281 (15)	2348 (18)	5173 (29)	2584 (35)		
≥18% and <20.7% (%)	1919 (23)	3004 (23)	4501 (26)	1728 (23)		
≥20.7% and <24.2% (%)	2487 (30)	3737 (29)	4048 (23)	1622 (22)		
≥24.2% (%)	2728 (32)	3845 (30)	3959 (22)	1528 (21)		<0.01^#^

Mean ± standard deviation or n (%)

*NGSP* National Glygohemoglobin Standardization Program, *IFCC* The International Federation of Clinical Chemistry and Laboratory Medicine

* Analysis results over 13 years; Additional file 1 shows this in detail

^#^ Using the Chi square test

^a^ Percentages in this column may not add up to exactly 100% because of rounding up

### The repeated cross-sectional study of antidiabetic prescription frequency

Table 2 shows extracted antidiabetic medication prescription data for changes in the prescription frequencies and daily doses of antidiabetic medications. An additional file shows this in detail (see Additional file 2). In total, 248,886 prescriptions were identified, for which data on 816,666 medications and treatments were identified, but we were not able to analyze 575 prescriptions (0.07%). After exclusion, data for 193,445 patients were analyzed. For biguanides, both the prescription frequency (21% in 2001 and 34% in 2013) and the daily dose (metformin rs = 0.36) increased. Notably, the prescription frequency of dipeptidyl peptidase-4 inhibitors increased significantly from 2% in 2010 to 38% in 2013, as did the daily dose (sitagliptin rs = 0.10; alogliptin rs = 0.14; and vildagliptin rs = 0.19). The daily dose of α-glucosidase inhibitors also increased (voglibose rs = 0.30 and acarbose rs = 0.04). By contrast, for sulfonylureas, both the prescription frequency (34% in 2001 to 29% in 2013) and daily dose of (glimepiride rs = −0.38) decreased significantly. In addition, the daily doses of meglitinides decreased slightly (nateglinide rs = −0.04 and mitiglinide rs = −0.12). More recently, the prescription frequency of thiazolidine decreased from 2011 onwards.

**Table 2:** The extracted antidiabetic medication prescription data obtained in the second repeated cross-sectional study

Year	2001	2005	2010	2013	rs^#^	p value^#^
n	14,996	13,976	17,561	3118		
*Sulfonylureas* (%)	5102 (34)	4016 (29)	5142 (29)	896 (29)		<0.01*
Glimepiride (n)	197	2414	4399	783		
Glimepiride (mg)	3 (2, 4) [0.5, 6]	2 (1, 3) [0.5, 6]	1 (0.5, 2) [0.5, 6]	1 (0.5, 1.5) [0.5, 6]	−0.38	<0.01
*Biguanide* (%)	3194 (21)	3633 (26)	5432 (31)	1050 (34)		<0.01*
Metformin (n)	3 164	3 622	5 430	1 050		
Metformin (mg)	750 (750, 750) [250, 1000]	750 (750, 750) [250, 1000]	750 (750, 750) [250, 1500]	750 (750, 1500) [250, 2250]	0.36	<0.01
*Thiazolidine* (%)	338 (2)	1907 (14)	6983 (40)	758 (24)		<0.01*
Pioglitazone (mg)	30 (30, 30) [15, 60]	15 (15, 30) [7.5, 30]	15 (15, 30) [7.5, 45]	15 (15, 30) [7.5, 45]	0.01	0.01
*α-GI (%)*	2936 (20)	2696 (19)	3966 (23)	561 (18)		<0.01*
Voglibose (n)	2351	2394	3390	493		
Voglibose (mg)	0.6 (0.6, 0.9) [0.2, 0.9]	0.6 (0.6, 0.9) [0.2, 0.9]	0.9 (0.6, 0.9) [0.2, 0.9]	0.9 (0.6, 0.9) [0.3, 0.9]	0.30	<0.01
*Meglitinides (%)*	654 (4)	1352 (10)	1418 (8)	155 (5)		0.45*
Nateglinide (n)	654	1275	1170	127		
Nateglinide (mg)	270 (270, 270) [60, 360]	270 (180, 270) [30, 360]	270 (180, 270) [30, 360]	270 (120, 270) [60, 360]	−0.04	<0.01
*DPP4I* (%)	0 (0)	0 (0)	361 (2)	1176 (38)		<0.01*
Sitagliptin (n)	0	0	361	818		
Sitagliptin (mg)			50 (50, 50) [25, 100]	50 (25, 50) [25, 100]	0.10	<0.01
*Insulins* (%)	3550 (24)	5085 (36)	5660 (32)	990 (32)		<0.01*

Median (interquartile range) [range] or n (%)

*α*-*GI* α-glucosidase inhibitors, *DPP4I* dipeptidyl peptidase-4 inhibitors

^#^ Analysis results over 13 years; Additional file 2 shows this in detail

* According to the Cochran-Armitage trend test

### The retrospective cohort study of visiting diabetes patients

In the retrospective study, we analyzed the data for 170 patients who continued to visit our hospital for diabetes treatment throughout the 13-year study period. We included patients for whom annual information was available about both diabetic control parameters and antidiabetic medication usage.

Table 3 shows extracted glycated hemoglobin and extracted glycated albumin data for annual changes in the mean values for diabetic control. An additional file shows this in detail (see Additional file 3). Similar to the results of the repeated cross-sectional studies, the mean values tended to decrease (HbA1c rs = −0.22, p < 0.01; glycated albumin rs = −0.14, p < 0.01). As for the four HbA1c categories, the proportion of patients with HbA1c levels >8% (>64 mmol/mol) decreased from 62% in 2001 to 31% in 2013, while the proportion of patients with HbA1c levels of 6 to <7% (42 to <53 mmol/mol) increased from 8% in 2001 to 27% in 2013. In addition, the proportion of patients with glycated albumin levels ≥22.0% fell from 66% in 2003 to 37% in 2013, and the proportion of patients with glycated albumin levels <22.0% increased from 35% in 2003 to 63% in 2013.

**Table 3:** The extracted glycated hemoglobin and extracted glycated albumin data obtained in the retrospective cohort study

Year	2001	2005	2010	2013	rs*	p value*
Evaluated HbA1c (NGSP) (n)	170	170	170	170		
HbA1c (NGSP)^a^ (%)	8.2 ± 0.9	8.0 ± 0.9	7.9 ± 1.0	7.7 ± 1.1	−0.22	<0.01
HbA1c (IFCC)^a^ (mmol/mol)	67 ± 10	64 ± 9	63 ± 11	60 ± 12		
HbA1c (NGSP) group^b^						
<6% (%)	0 (0.0)	0 (0.0)	1 (1)	1 (1)		
≥6% and <7% (%)	14 (8)	15 (9)	21 (12)	45 (27)		
≥7% and <8% (%)	51 (30)	73 (43)	79 (47)	72 (42)		
≥8% (%)	105 (62)	82 (48)	69 (41)	52 (31)		<0.01^#^

Year	2003	2005	2010	2013	rs*	p value*
Evaluated glycated albumin (n)	136	170	170	168		
Glycated albumin^a^ (%)	23.5 ± 3.6	22.8 ± 3.5	22.3 ± 3.9	21.5 ± 4.4	−0.14	<0.01
Glycated albumin quartile^b^						
<19.7% (%)	20 (15)	28 (17)	45 (27)	59 (35)		
≥19.7% and <22.0% (%)	27 (20)	52 (31)	45 (27)	47 (28)		
≥22.0% and <24.6% (%)	42 (31)	40 (24)	43 (25)	26 (16)		
≥24.6% (%)	47 (35)	50 (29)	37 (22)	36 (21)		<0.01^#^

Mean ± standard deviation or n (%)

*NGSP* national glygohemoglobin standardization program; *IFCC* the international federation of clinical chemistry and laboratory medicine

* Analysis results over 13 years; Additional file 3 shows this in detail

^#^ Using the Chi square test

^a^Yearly mean value for each patient

^b^Percentages in this column may not add up to exactly 100% because of rounding up

Table 4 shows extracted antidiabetic medication prescription data for the prescription frequencies and median daily doses, calculated annually, of the antidiabetic medications identified in the retrospective cohort. An additional file shows this in detail (see Additional file 4). The prescription frequency of dipeptidyl peptidase-4 inhibitors increased from 1% in 2010 to 41% in 2013. The median daily doses of α-glucosidase inhibitors and biguanides increased per year during the study period (voglibose rs = 0.26; metformin rs = 0.28). As for sulfonylureas, the median daily dose (glimepiride rs = −0.45) and mean prescription rate (46% in 2001 and 35% in 2013) both decreased annually. The yearly median daily dose of meglitinides also decreased (nateglinide rs = −0.18), and the prescription frequency of thiazolidine decreased from 2012 onwards.

**Table 4:** The extracted antidiabetic medication prescription data obtained in the retrospective cohort study

Year	2001	2005	2010	2013	rs^#^	p value^#^
n	170	170	170	170		
*Sulfonylureas (%)*	78 (46)	63 (37)	67 (39)	59 (35)		0.02*
Glimepiride (n)	12	31	54	47		
Glimepiride^a^ (mg)	3 (2, 5.625) [1, 6]	3 (2, 4) [1, 6]	1.875 (1, 3) [0.5, 6]	1 (0.5, 2) [0.5, 6]	−0.45	<0.01
*Biguanide* (%)	78 (46)	83 (49)	84 (49)	76 (45)		0.76*
Metformin (n)	77	83	84	76		
Metformin^a^ (mg)	750 (750, 750) [375, 750]	750 (750, 750) [250, 750]	750 (750, 750) [250, 1000]	750 (750, 1500) [250, 2250]	0.28	<0.01
*Thiazolidine* (%)	5 (3)	35 (21)	83 (49)	48 (28)		<0.01*
Pioglitazone^a^ (mg)	30 (30, 30) [30]	15 (15, 30) [7.5, 30]	15 (15, 30) [7.5, 45]	15 (15, 30) [7.5, 45]	0.06	0.16
*α-GI (%)*	54 (32)	56 (33)	60 (35)	43 (25)		0.30*
Voglibose (n)	43	50	52	37		
Voglibose^a^ (mg)	0.9 (0.6, 0.9) [0.4, 0.9]	0.6 (0.6, 0.9) [0.2, 0.9]	0.9 (0.6, 0.9) [0.2, 0.9]	0.9 (0.6, 0.9) [0.3, 0.9]	0.26	<0.01
*Meglitinides (%)*	12 (7)	15 (9)	22 (13)	8 (5)		0.44*
Nateglinide (n)	12	15	19	6		
Nateglinide^a^ (mg)	270 (270, 270) [270, 270]	270 (270, 270) [60, 270]	270 (180, 270) [60, 360]	135 (90, 225) [90, 360]	−0.18	0.01
*DPP4I (%)*	0 (0)	0 (0)	1 (1)	70 (41)		<0.01*
Sitagliptin (n)	0	0	1	52		
Sitagliptin^a^ (mg)			50 (50, 50) [50, 50]	50 (25, 50) [25, 100]	0.09	0.30
*Insulins (%)*	69 (41)	96 (57)	97 (57)	93 (55)		<0.01*

Median (interquartile range) [range] or n (%)

*α*-*GI* α-glucosidase inhibitors, *DPP4I* dipeptidyl peptidase-4 inhibitors

^#^ Analysis results over 13 years; Additional file 4 shows this in detail

* According to the Cochran-Armitage trend test

^a^Yearly median daily dose for each patient

## Discussion

In the present study, we conducted two large-scale repeated cross-sectional studies and a long-term retrospective cohort study to examine the long-term changes in prescribing antidiabetic medications and to examine the associated changes in diabetic control. First, we demonstrated, through changes in parameters of glycemic control, that diabetes management improved over the study period. In addition, we showed the characteristics of changes in antidiabetic prescribing at our institution over a 10-year period. To the best of our knowledge, this is the first large-scale, long-term observational study of diabetes based on electronic medical records in Japanese patients.

We obtained evidence that parameters of long-term diabetic control improved, consistent with the results of a previous multicenter prospective study by the Japan Diabetes Clinical Data Management Study Group (JDDM), which revealed a tendency toward improved HbA1c levels [19]. In 2014, the JDDM had 98 participating institutions in Japan and had approximately 74,000 registered patients with diabetes. However, other long-term interventional studies have failed to detect any tendency toward improvement in HbA1c levels [20–23]. Similar to the repeated cross-sectional studies carried out here, the JDDM study was also a repeated cross-sectional study. However, such cross-sectional studies are limited in their ability to estimate the long-term management statuses of individual patients, while the intervention studies were limited to the available methods for treating eligible patients and had HbA1c targets set in advance. Accordingly, the results of these studies cannot readily be applied to clinical practice.

The present research included a retrospective cohort study to assess changes in patients over the 13-year study period. In addition, we included glycated albumin levels. By examining glycated albumin levels, our assessment of changes in management was also based on two parameters. Therefore, the present results provide more useful estimates of the long-term trends in clinical diabetes management.

The present study showed that there were large changes in the prescription of antidiabetic medications over the 13-year study period. Changes in diabetes management guidelines and several factors may explain the changes seen in prescription patterns during the study period. First, in the mid-2000s, metabolic syndrome and the recommendation of the guidelines seemed to promote prescriptions of thiazolidine and α-glucosidase inhibitors and increase the daily dose of voglibose [11–13]. Second, the daily dose of glimepiride and meglitinides showed a tendency to decrease around 2010. Previous studies have shown that severe hypoglycemia is associated with health risks during the treatment of diabetes [24–26]. It is therefore recommended that during pharmaceutical treatment for diabetes, treatment strategies should be employed to avoid unnecessary hypoglycemia [14, 27]. One study on antidiabetic medication prescription rates in the USA showed trends toward increases in the prescription rates of biguanides and dipeptidyl peptidase-4 inhibitors, with a concurrent trend toward reductions in the prescription rates of sulfonylureas [28], consistent with the findings of the present study. Monotherapy with biguanides or dipeptidyl peptidase-4 inhibitors is associated with a lower risk of inducing hypoglycemia than sulfonylurea treatment. Attempts to reduce the risk of hypoglycemia may account for similar trends in prescription rates in Japan and the USA. After 2010, the prescription rates of dipeptidyl peptidase-4 inhibitors increased, and the daily doses of metformin increased. On the other hand, the prescription rates of thiazolidine decreased. Dipeptidyl peptidase-4 inhibitors were introduced clinically in Japan in 2009, while the maximum insurance-covered dose of metformin was increased from 750 to 2250 mg/day in 2010. Recent studies have also suggested that thiazolidine increases the risk of bladder cancer and has negative effects on bones [29, 30]. In 2012, dipeptidyl peptidase-4 inhibitors were added to the second line of diabetes treatment [14]. Changes in guidelines and these factors may explain several observed trends after 2010. Although the tendency to increase the prescription rates was shown in the repeated cross-sectional study, we were interested in the lower prescription rates of biguanide. We thought that this issue was due to Japanese guidelines and a characteristic of Japanese patients with diabetes. Since the 2000s, Japanese diabetes treatment guidelines recommend that physicians provide care in accord with an individual patient’s status. These guidelines do not designate the first-line drug of diabetes pharmacotherapy [18]. In addition, as is widely known, it is thought that one of the major cause of Japanese diabetes is a deficiency of insulin secretion. These factors may explain the low prescription rates of biguanide.

Our study had several limitations. First, we could not confirm whether participants had firm diagnoses of diabetes and visited only our institution for treatment over the 13 years of this study. We defined patients as having diabetes if they visited the Endocrinology and Metabolism Division, had a recorded diagnosis of E10 to E14 according to the ICD-10, and had their HbA1c levels measured. However, because this was a retrospective study based on medical records, we do not know if these patients actually met the diagnostic criteria for diabetes. In addition, not all patients who had their HbA1c levels measured may have attended our institution regularly for diabetes management. However, the measurement of HbA1c is not approved in Japan for individuals without diabetes or where there is no clinical suspicion of diabetes. Similarly, the prescription of antidiabetic medications has not been approved for patients without diabetes. Therefore, we are confident that almost all of the participants in our analysis had diabetes.

Second, the causal relationships between changes in the use of antidiabetic medications and variations in the parameters of diabetic control remain unclear. Indeed, this was only an observational study, so we cannot infer which treatments improved diabetic control. We also did not investigate insulin prescriptions in detail. Therefore, further prospective studies are needed to analyze which factors help improve diabetic control.

Third, the results of the present study have limited generalizability. This was only an observational study based on the data from a single institution. In addition, our “visiting diabetes patients” included only very compliant patients who had survived 13 years of diabetes, which introduced bias in the results. Therefore, at best, the results are limited in their applicability to extremely compliant patients with diabetes in Japan.

## Conclusions

The present study revealed a tendency toward long-term improvements in indicators of diabetic control and revealed that there were major changes in the prescribing frequencies of the most common antidiabetic medications at our institution. However, these changes were consistent with changes in guideline recommendations over the study period. We conclude that new antidiabetic medications and treatment strategies might have positive effects on diabetes management but that further multicenter research is needed.

## Additional files

**Additional file 1: Table S1.** The glycated hemoglobin and glycated albumin data in the first repeated cross-sectional study.

**Additional file 2: Table S2.** The antidiabetic medication prescription data obtained in the second repeated cross-sectional study.

**Additional file 3: Table S3.** The glycated hemoglobin and glycated albumin data obtained in the retrospective cohort study.

**Additional file 4: Table S4.** Antidiabetic medication prescription data obtained in the retrospective cohort study.

## Abbreviations

HbA1c: glycated hemoglobin
ID: identification
JDS: Japan Diabetes Society
IFCC: International Federation of Clinical Chemistry
NGSP: national glycohemoglobin standardization program
ICD: international statistical classification of diseases and related health problems
JDDM: The Japan Diabetes Clinical Data Management Study Group
